# Gene Expression-Based Classification of Non-Small Cell Lung Carcinomas and Survival Prediction

**DOI:** 10.1371/journal.pone.0010312

**Published:** 2010-04-22

**Authors:** Jun Hou, Joachim Aerts, Bianca den Hamer, Wilfred van IJcken, Michael den Bakker, Peter Riegman, Cor van der Leest, Peter van der Spek, John A. Foekens, Henk C. Hoogsteden, Frank Grosveld, Sjaak Philipsen

**Affiliations:** 1 Cell Biology, Erasmus University Medical Center, Rotterdam, The Netherlands; 2 Cancer Genomics Center, Erasmus University Medical Center, Rotterdam, The Netherlands; 3 Pulmonary Diseases, Erasmus University Medical Center, Rotterdam, The Netherlands; 4 Center for Biomics, Erasmus University Medical Center, Rotterdam, The Netherlands; 5 Pathology, Erasmus University Medical Center, Rotterdam, The Netherlands; 6 Bioinformatics, Erasmus University Medical Center, Rotterdam, The Netherlands; 7 Medical Oncology, Erasmus University Medical Center, Rotterdam, The Netherlands; Queen Elizabeth Hospital, Hong Kong

## Abstract

**Background:**

Current clinical therapy of non-small cell lung cancer depends on histo-pathological classification. This approach poorly predicts clinical outcome for individual patients. Gene expression profiling holds promise to improve clinical stratification, thus paving the way for individualized therapy.

**Methodology and Principal Findings:**

A genome-wide gene expression analysis was performed on a cohort of 91 patients. We used 91 tumor- and 65 adjacent normal lung tissue samples. We defined sets of predictor genes (probe sets) with the expression profiles. The power of predictor genes was evaluated using an independent cohort of 96 non-small cell lung cancer- and 6 normal lung samples. We identified a tumor signature of 5 genes that aggregates the 156 tumor and normal samples into the expected groups. We also identified a histology signature of 75 genes, which classifies the samples in the major histological subtypes of non-small cell lung cancer. Correlation analysis identified 17 genes which showed the best association with post-surgery survival time. This signature was used for stratification of all patients in two risk groups. Kaplan-Meier survival curves show that the two groups display a significant difference in post-surgery survival time (p = 5.6E-6). The performance of the signatures was validated using a patient cohort of similar size (Duke University, n = 96). Compared to previously published prognostic signatures for NSCLC, the 17 gene signature performed well on these two cohorts.

**Conclusions:**

The gene signatures identified are promising tools for histo-pathological classification of non-small cell lung cancer, and may improve the prediction of clinical outcome.

## Introduction

Lung cancer is the most frequent cause of cancer deaths in the North America and Europe. In Europe alone, there were 386,300 new lung cancer cases in 2006, with an estimated 334,800 deaths. This accounts for 13.5% of all cancer deaths [1]. Based on histo-pathological presentation, lung cancer is sub-divided into four major histological subtypes: small cell lung cancer (SCLC), squamous cell carcinoma (SCC), adenocarcinoma (ADC), and large cell carcinoma (LCC). The latter three, collectively referred to as non-small cell lung cancer (NSCLC), account for almost 80% of lung cancers [2]. At present, treatment of NSCLC is based on histo-pathological features and staging. However, pathologically similar tumors with comparable stage show dramatically different response to the same therapy. Common features at the molecular level may be able to predict such outcome discrepancies among patients more reliably. For instance, the efficacy of epidermal growth factor receptor (EGFR) antagonists has been shown to depend on expression of its target -EGFR- in the tumor [3]. Also, the beneficial effect of chemotherapies might depend on the expression of certain proteins such as thymidine synthetase for Pemetrexed [4]. Thus, improved classification of NSCLC is of considerable clinical interest.

Recent advances in microarray technology enable researchers to recapitulate molecular properties of NSCLC at the level of individual genes [5], [6], [7], [8], [9]. However, the reproducibility of gene expression signatures to predict high-risk of relapse or recurrence is rarely reported. Therefore, it is highly desirable to identify molecular classifiers that can reliably predict specific subgroups of high- and low-risk patients. This would be helpful to select the most appropriate therapy for individual patients.

In this study, we performed gene expression profiling on NSCLC tumors and simultaneously collected normal lung tissue samples in order to determine histo-pathological classifier genes and high-risk index genes.

## Materials and Methods

A detailed description is provided in File S1.

### Patient enrolment

Ninety-one NSCLC patients treated at the Erasmus MC were included in this study. The written consent from all participants involved in this study was obtained. Patient and tumor characteristics are listed in Table 1. Tissues were studied under an anonymous tissue protocol approved by the medical ethical committee of Erasmus University Medical Center.

**Table 1:** Characteristics of patients and samples.

			Training set			Validation set	
			(N = 80)			(N = 76)	
Heath			36			29	
Tumor			44			47	
Mean age (years)			62.3±10.81			63.5±10.73	
Sex-%	Female		27			34	
	Male		73			66	
Race-%	Caucasian		90			89	
	other		5			3	
	unknown		5			8	
Tobacco history-%	None		-			-	
	≤30 yr		20			24	
	31–49 yr		20			18	
	≥50 yr		18			18	
	unknown		41			39	
Tumor type (n)	Path. Review	1st	2^nd^	consistent	1st	2nd	consistent
	ADC	19	14	14	13	10	8
	SCC	16	8	8	11	8	8
	LCC	7	13	6	6	11	3
	other	2	9	1	8	9	1
	unknown	0	0		9	9	
Stage-%	Path. Review	1st			1st		
	IA	18			16		
	IB	45			42		
	IIA	2			-		
	IIB	30			21		
	IIIA	2			16		
	IIIB	-			-		
	IV	2			5		
Status-%	Alive		34			29	
	Deceased		61			63	
	unknown		5			8	
Cause of death-%	Lung cancer		27			34	
	other		18			18	
	unknown		55			47	

We used two independent validation sets: 6 normal lung tissues from GSE3526, and NSCLC samples from the Duke University cohort [10].

### Pathological analysis

Tumor samples were typed by two independent routine pathological reviews, according to WHO guidelines [11]. Histochemical stains (periodic acid-Schiff and Alcian blue for mucin) were applied when considered appropriate.

### RNA Isolation and gene expression profiling

Dissected tumors and adjacent normal tissue were snap-frozen in liquid nitrogen precooled isopentane within two hours after surgical resection, and stored at −196°C or −80°C until RNA extraction. 5 ìg of total RNA was processed for analysis on Affymetrix U133 plus 2.0 arrays using standard protocols. The complete microarray data is MIAME compliant and deposited in a MIAME compliant database, Gene Expression Omnibus database at www.ncbi.nlm.nih.gov/geo/info/linking.html (GSE19188).

### Bioinformatics analyses

Multiple parameters were used to control the overall quality of arrays. The final intensity value of probe sets was summarized as the deviation to the geometric mean of that probe set among all arrays. Uninformative probe sets were eliminated and the remaining probe sets were used for subsequent analyses.

### Class comparison

Two-group comparisons were performed by Significance Analysis of Microarrays [12]. This supervised analysis correlates gene expression with a clinical variable based on a score calculated using the change in expression and the standard deviation across all samples.

### Class prediction

All identified signatures were subjected to identify subgroups of genes that maintain the capacity of the complete signatures in distinguishing different groups maximally [13]. The performance of optimized signatures was validated by “leave-one-out” cross validation within the training set firstly, then with the validation set [14]. Hierarchical clustering was performed using the Spotfire Decision Site.

### Survival analysis

We developed a step-wise approach based on gene expression profiles to classify NSCLC with respect to prognostic outcome. Firstly, the Wald test in the Cox proportional hazards model was used to identify prognostic probe-sets which were the most likely associated with overall survival [15]. Candidate probe sets were selected based on p-values (<0.001) computed from 1000 random permutations. The resulting candidate survival probe sets were subjected to a supervised analysis [16], which comprises computation of principal components with candidate probe sets, Cox proportional hazards regression analysis using the resulting principal components, and finally prognostic predictor calculation by fitting the predictive prognosis model derived from the Cox regression. The predictive value of the prognosis model was evaluated by performing “leave-one-out” cross-validation [16], [17]. The prognostic value of the prognostic predictor relative to clinical variables, such as age, tumor cell content (%), tumor size (diameter of tumor), smoking year, Forced Expiratory Volume 1, gender, histology, and tumor grade was tested by the Wald test (Table S8). The correlation between the survival signature and clinical parameters is summarized in Table S7.

### Other NSCLC classifiers

The signatures identified in this study were compared to published histology and prognosis signatures. The tested histology signatures were derived from Affymetrix U95A chips {[18] and US20040241725A1}, IntelliGene chips [9], and Stanford cDNA oligonucleotide arrays [19] (Table S9). The survival related signatures were 20- and 6-probe set predictors developed by Lee et al [20], one signature derived from Affymetrix U133A chips [21], one from Affymetrix HuGeneFL chips [22], [23], two from other types of oligonucleotide array [24], [25], and one from RT-PCR assays [26] (Table S10).

## Results

### Study design

**Figure 1:**
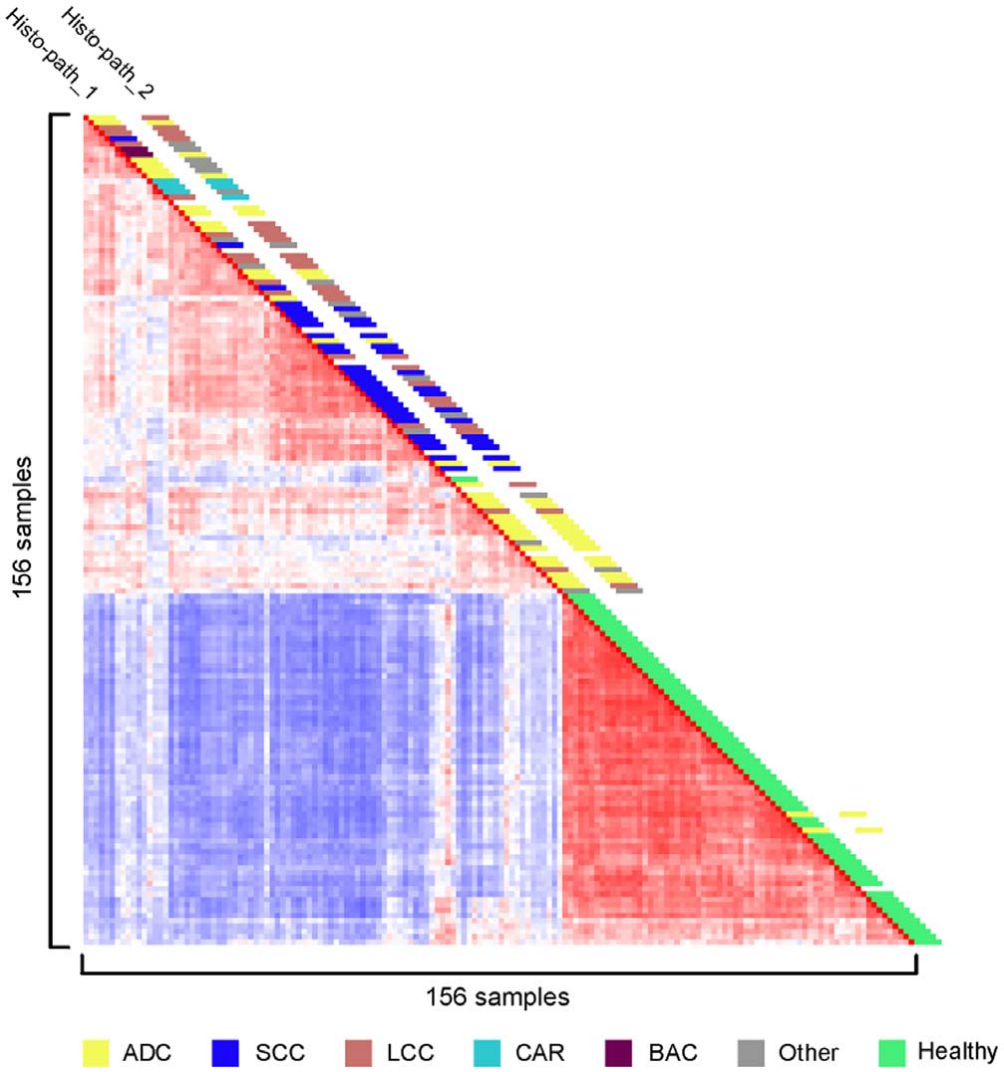
Correlation view of 156 samples from patients with NSCLC. Pairwise correlations between any two samples are displayed, based on 4791 informative probe sets. The colors of the cells represent Pearson's correlation coefficient values, with deeper red indicating higher positive and deeper blue lower negative correlations. The red diagonal line displays the self-to-self comparison of each sample. Histological classification of the samples is depicted along the diagonal; the key to the color code is shown at the bottom. Histo-path_1 & Histo-path_2: initial and second histo-pathological review.

Tumors (n = 91) and unaffected lung tissue samples (n = 65) were collected from NSCLC patients undergoing lung resection at Erasmus MC between 1992 and 2004. The tissue specimens were snap frozen in liquid nitrogen pre-cooled isopentane and stored in liquid nitrogen or at −80°C until further processing. The clinical parameters of the patients enrolled in this study are summarized in Table 1. Paraffin sections of the tumors were scored by routine pathology and an independent pathologist (MdB) for histo-pathological characteristics. Eight LCC samples had a high level of cell type heterogeneity, presenting with acinar differentiation and squamous cell components. Eighteen samples had a discrepancy in histopathological classification (Table 1 and Fig. 1), including five representing rare types of NSCLC with a histological composition of multiple cell types. We isolated RNA from 25 µm cryostat sections of the snap-frozen specimens and used this for labelling and hybridisation to Affymetrix U133 2.0 plus arrays. Tumor cell content was determined from 10 µm sections taken at the start and end of cryostat cutting. The samples were divided into two sets, training and validation (Table 1), according to the criteria presented in File S1, and used for the subsequent bioinformatics analyses. By unsupervised Pearson's correlation analysis, tumor samples were clearly separated from the healthy lung samples (Fig. 1). We therefore first sought to derive a minimized signature gene set that could distinguish tumors from healthy lung tissue.

### Signature genes distinguish NSCLC from normal lung tissue

**Figure 2:**
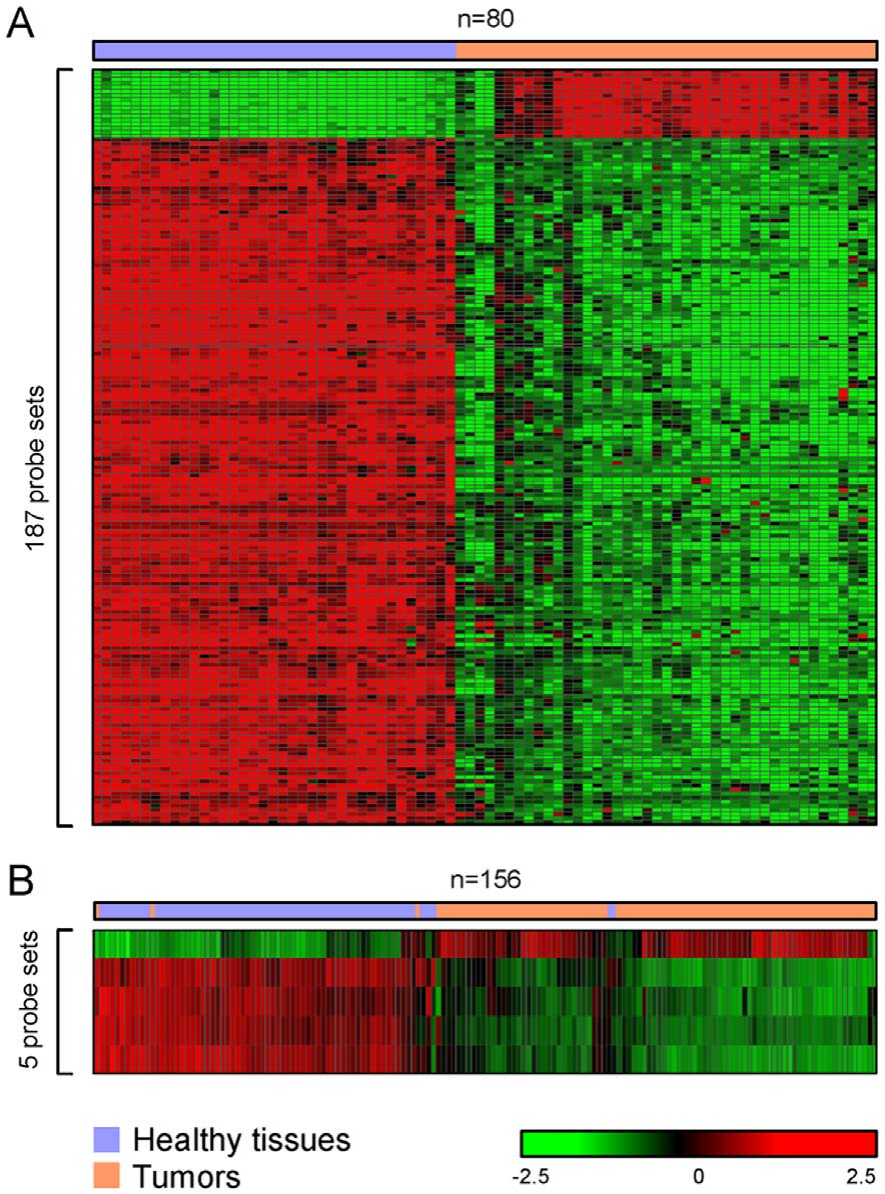
Hierarchical clustering distinguishes tumors from healthy lung tissue. **A**: Two-dimensional hierarchical clustering of 80 training samples, including tumors and healthy lung samples, was performed with 187 probe sets. The relative expression to the overall mean for each probe set (rows) in each sample (columns) is indicated by a color code. **B**: Hierarchical clustering of 156 tissue samples with 5 probe sets yields 2 groups, tumor and normal lung.

To identify a signature gene set for NSCLC tumors, we compared gene expression profiles from 44 tumors with that from 36 healthy lung tissues. Histology-driven analyses generated in total 415 common probe-sets presenting differential expression in three major types of NSCLC (data not shown). To find genes more generally expressed by all NSCLC cases, all tumors were compared to all healthy lung tissues without taking histological information into account. By using different cut-offs in supervised analysis, we identified sets of thousands to hundreds of probe-sets characterizing NSCLC (Table S1). A final list of 187 probe sets that were differentially expressed in all NSCLC samples was determined as the Tumor Signature (Fig. 2A and Table S2). A subset of these probe sets, 5 out of 187, was able to distinguish non-cancerous tissues from malignant NSCLC with an accuracy of 98%, using Prediction Analysis of Microarrays (Fig. 2B and Table S3). Two tumor and three non-cancerous lung tissue samples were incorrectly classified by the optimized tumor signature. Of these, one presented with an uncertain histological diagnosis, and two were from patients who had developed multiple primary tumors. We conclude that the expression signature of these 5 probe sets accurately distinguishes NSCLC from healthy lung tissue, regardless of NSCLC subtype.

#### NSCLC are sub-classified by histology signature genes

**Figure 3:**
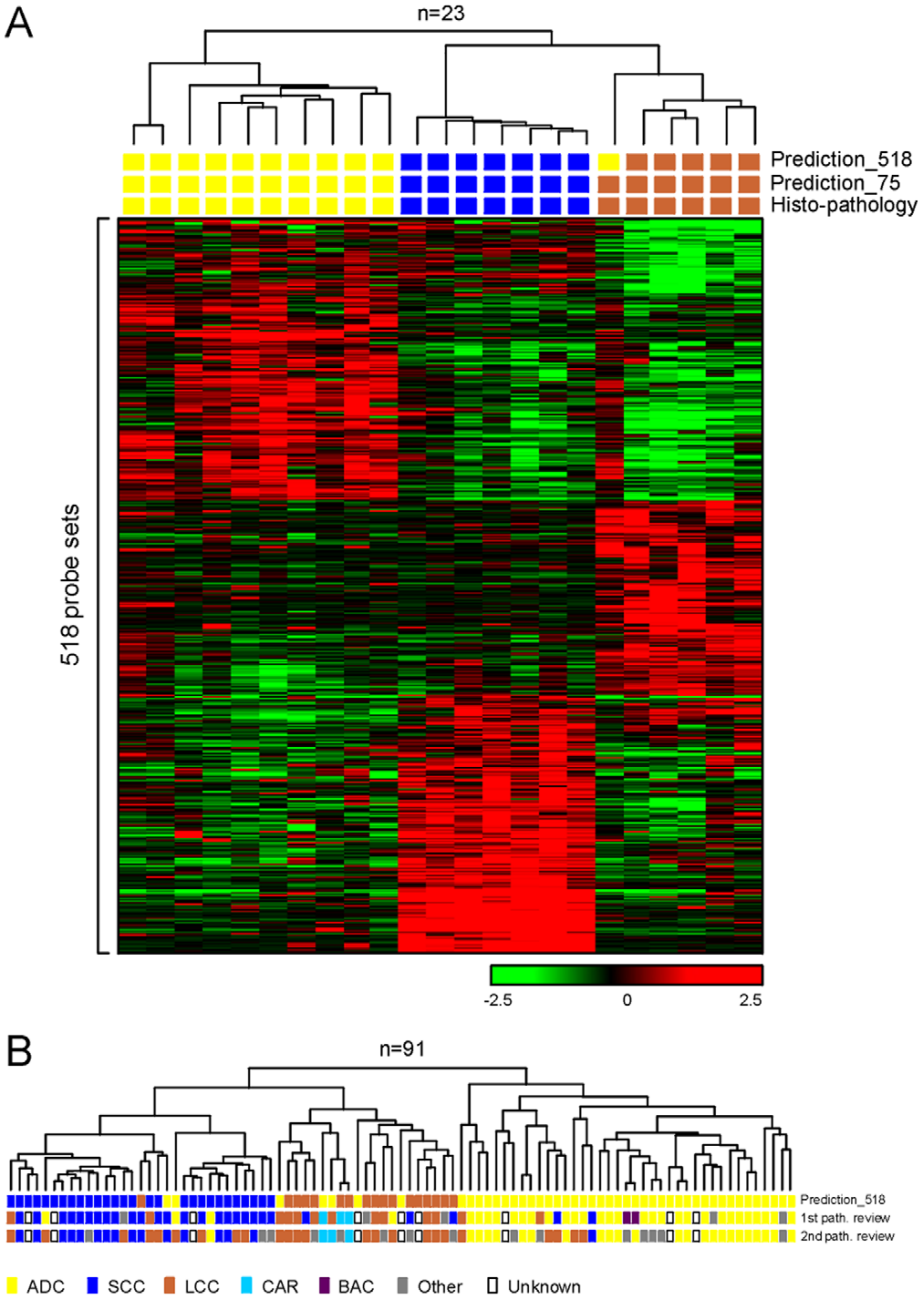
Clustering analysis of NSCLC tumors with the 518 probe set histology signature. **A**: agglomerative hierarchical clustering of 23 NSCLC samples using the 518 probe set histology signature. The relative expression to the overall mean for each probe set (rows) in each sample (columns) is indicated by a color code. Correlation between the samples is depicted by the dendrogram. Histo-pathological diagnosis and predictions of histology subtype by Prediction Analysis of Microarrays, using the 518 and 75 probe set signatures, are shown by colored blocks. **B**: correlation dendrogram generated by agglomerative hierarchical clustering of all 91 Erasmus MC NSCLC samples using the 518 probe set signature. Histo-pathological diagnosis of the initial and second review, and prediction of histology subtype by Prediction Analysis of Microarrays using the 518 probe set signature, are shown by colored blocks.

**Figure 4:**
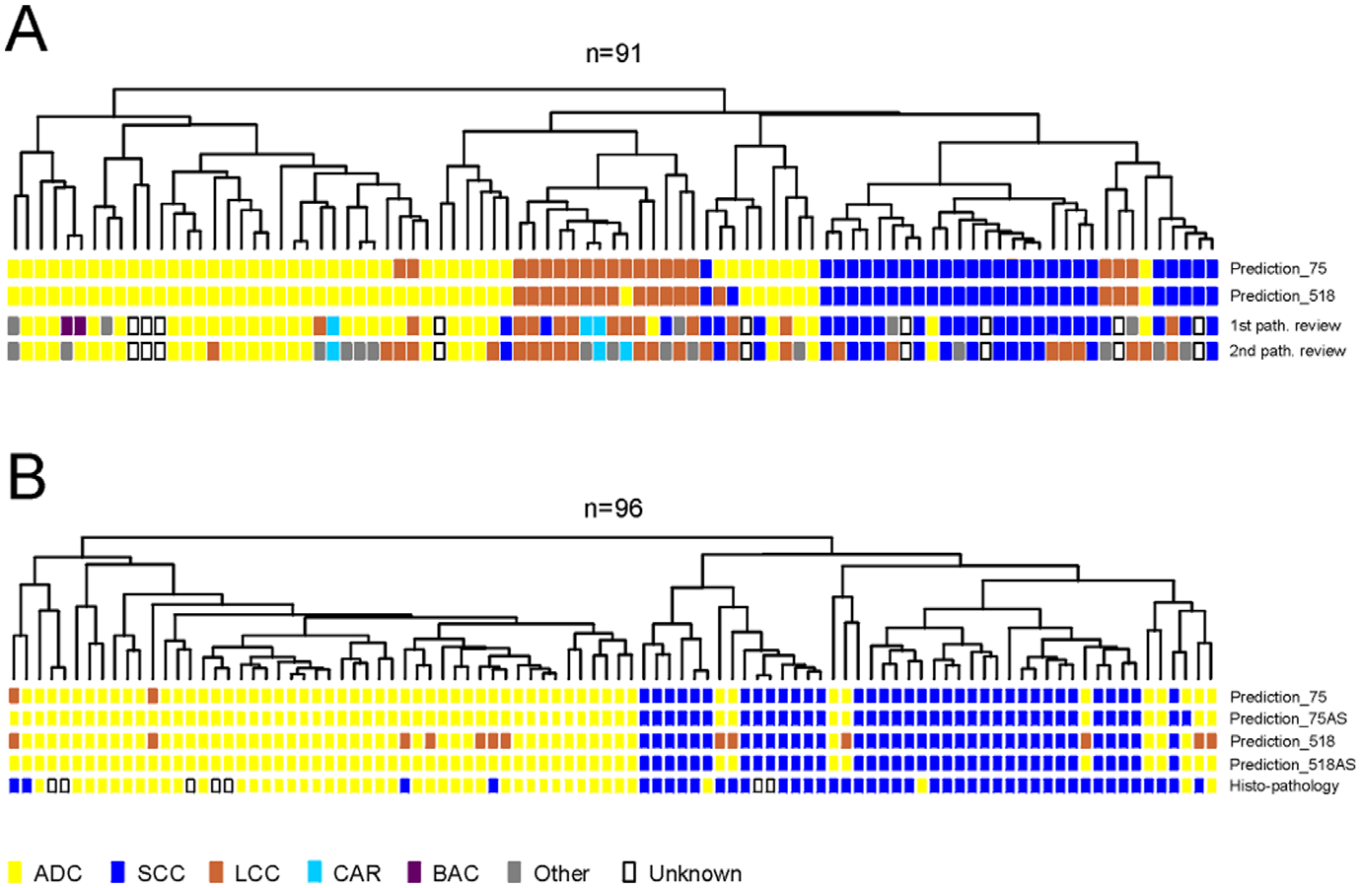
Prediction of histology subtype of Erasmus MC and Duke University NSCLC samples. **A**: correlation dendrogram generated by agglomerative hierarchical clustering of all 91 Erasmus MC NSCLC samples using the 75 probe set histology signature. Histo-pathological diagnosis of the initial and second review, and prediction of histology subtype by Prediction Analysis of Microarrays using the 75- and 518 probe set histology signatures, are shown by colored blocks. **B**: correlation dendrogram generated by agglomerative hierarchical clustering of all 96 Duke University NSCLC samples using the 75 probe set histology signature. The reported histo-pathological diagnosis, and prediction of histology subtype by Prediction Analysis of Microarrays using the 75- and 518 probe set histology signatures, are shown by colored blocks. 75AS and 518AS: prediction without the LCC probe sets in the histology signatures, using 68 and 329 probe sets respectively (see Tables S4 and S5).

As NSCLC are tumors with a high degree of heterogeneity, genes characterizing histological features were identified using strictly selected tumor samples. Firstly, the histological diagnosis had to be consistent between the two independent pathology reviews. Secondly, the samples should not display apparent tumor cell heterogeneity. Thirdly, the content of cancer cells should be above 60%. We compared the gene expression profiles of each NSCLC subtype to those of the other two subtypes, and identified a total of 518 probe sets representing the three major subtypes of NSCLC – ADC, SCC, and LCC (Table S4). Using “leave-one-out” cross validation, we found that the percentage of correct classification by Prediction Analysis of Microarrays was 96% (22 out of 23) in the training samples (Fig. 3A). When this signature was applied to classify the validation samples, we found that the three carcinoid (CAR) samples, which were not involved in deriving the signature, and one LCC sample were separated from the other tumors by clustering with the 518 probe sets, thus representing a unique group (Fig. 3B). We note that the LCC sample in this group was classified as CAR by the second pathology review. The optimized signature gene set consisted of 75 probe sets (Table S5). This optimised signature classified the training samples with 100% accuracy (Fig. 3A). The expression profile of those genes was applied to predict the histology subtype of the samples with conflicting pathology diagnoses (n = 18). With three exceptions, all the ambiguously classified LCCs (n = 11) were determined as ADC or SCC by the optimized gene signature, and this was consistent with the primary diagnosis (Fig. 4A). Of the 18 samples, one had an ambiguous diagnosis due to unsatisfactory histology, and three had a tumor cell content of less than 20%. We note that over 60% (n = 11) of these 18 samples presented with apparent tumor cell type heterogeneity. Our results suggest that the 75 probe set histology signature may aid in assigning the correct histological classification in ambiguous cases of NSCLC.

### Survival risk prediction by expression profiles

To derive prognostic information from the gene expression profiles, we first divided NSCLC patients into groups with either short (<2 years) or long (>5 years) overall survival. Comparing the profiles of these two groups failed to identify any significant differences in gene expression with a false discovery rate <20%. Similar negative results were obtained when the analyses were restricted to ADC or LCC cases. A set of 29 probe sets was identified with differential expression between SCC patients with short- and long survival (false discovery rate <10%; data not shown).

**Figure 5:**
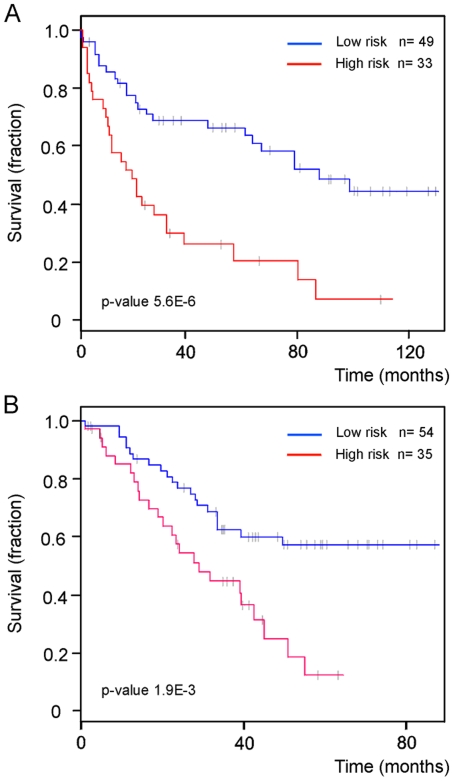
A 17 probe set signature predicts patient survival time. Kaplan-Meier curves for **A**: 82 Erasmus MC NSCLC patients and **B**: 89 Duke University NSCLC patients fitted by their risk assignments based on the 17 probe set survival signature. The high- and low-risk groups differ significantly, indicated by the p-values. Grey bars indicate patients at last follow-up, still alive.

Starting with the 11,515 probe sets remaining from the data filtering process, we identified a subset of informative probe sets that were best correlated with survival time using the Wald test from the Cox proportional hazards model. The principal components computed from the expression of these genes were subjected to Cox proportional hazard regression analysis, and built up a model for predicting a prognostic probability for each NSCLC case. The predictive value of the prognosis model was evaluated and optimized by performing “leave-one-out” cross-validation, and resulted in an optimized model consisting of 17 probe sets. The survival signature included the EGFR gene, a prominent gene contributing to prognosis variation in diverse solid tumor such as breast and colorectal cancer [27], [28], [29], [30] (Table S6). A risk percentile cut-off of 60% was used to define two risk groups, which were distinguished at significance p-value = 5.6E-6 by log-rank test. A Kaplan-Meier curve of overall survival from these two risk groups is shown in Fig. 5A.

The association between the prognosis profile and clinical parameters was studied. The prognosis profile was significantly associated with age (p<0.023), smoking years (p<0.014), gender (p<0.012) and Forced Expiratory Volume 1 (p<0.009), a parameter reflecting lung function, but not with tumor stage, tumor cell content, tumor histology and tumor size (Table S7). We performed multivariate proportional hazard regression analysis to evaluate the predictive value of the prognostic predictor for patient outcome in comparison with other clinical parameters. No evidence of relation was found between relative hazard ratio and age, gender, smoking year, tumor cell content, Forced Expiratory Volume 1, tumor histology and tumor size. Table S8 shows the Wald statistics and significance for each variable tested. Tumor stage and the 17 probe set prognostic predictor were significantly related to the hazard of death. However, the prognostic predictor presented the highest importance which was 21.682 compared to 3.797 from tumor stage. Moreover the relative hazard ratio predicted by the prognostic predictor was 2.465 (95% confidence interval, 1.686 to 3.604, p<1.5E-06), the highest one among all tested risks (Table 2). Similarly, the inclusion of the prognostic predictor to the predictive model resulted in a change in model performance of 19.5, in terms of −2 log likelihood, with a p-value of 9.8E-06, compared to 24.3 and 2.0E-03 introduced by the model comprising all clinical variables. Thus, the multivariate proportional hazard analysis strongly indicates that the gene expression profile-derived prognostic predictor is the strongest predictor of the likelihood of death.

**Table 2:** Multivariable proportional hazard analysis of the risk of death.

		HAZARD RATIO	Change in -2 log likelihood	Significance
		(95% confidence interval)		
**Age**	1.03	(0.99–1.07)	10.35	0.001293
**Tumor cell %**	1.01	(0.99–1.03)	2.16	0.141500
**Stage**	1.32	(1.00–1.74)	3.90	0.048425
**Gender**	1.00	(0.44–2.27)	2.78	0.095444
**Smoking years**	1.00	(0.97–1.04)	1.13	0.286797
**Forced Expiratory Volume 1**	1.01	(0.99–1.03)	0.51	0.476836
**Tumor size**	1.00	(0.98–1.03)	0.00	0.979352
**Histology**	0.91	(0.81–1.02)	3.49	0.061814
**Prognostic predictor**	2.47	(1.69–3.6)	19.55	0.000010

### Validation of signature probe sets

We studied the expression patterns of all signatures in two independent sets of microarray data collected in the United States (US validation set), a subset of the NSCLC cohort from Duke University (n = 96) [10], and 6 normal lung specimens from GSE3526 (NCBI GEO database). These were chosen because 1) they were also analyzed on the Affymetrix U133 plus 2.0 arrays, and 2) the original .CEL files were available (i.e. raw rather than pre-normalized data). The optimized 5 probe set tumor signature performed on the US validation set with an accuracy of 97%: 93 out of 96 NSCLC were correctly classified as ‘tumor’ and all normal lung specimens were correctly classified as ‘healthy’. Since there were no LCC or other types of NSCLC in the Duke University data set, we only used the ADC and SCC signature probe sets, comprising 68 of the 75 probe sets in the histology signature (Tables S4 and S5), for histological classification of the Duke University NSCLC samples. For 84% of Duke University samples, the prediction by the 68 probe set ADC/SCC signature was consistent with the reported histology diagnosis. When the LCC signature was included in the prediction analysis, this percentage decreased to 83%: 2 samples were classified as LCC (Fig. 4B). Follow-up data were available for 89 of 96 patients in the Duke University cohort, and we calculated the prognostic predictor for these patients using the 17 probe set survival signature and the predictive model. The difference in the hazard of death between the patient groups with a predicted good prognosis and the group with a poor prognosis was 2.44-fold, with a significance of p-value = 1.9E-03 by log-rank test. A Kaplan-Meier curve of overall survival is shown in Fig. 5B. If the Erasmus MC patient cohort is combined with the cohort recruited at Duke University, the p-value reduces to 2.6E-7 (data not shown).

### Comparison with published histology and prognostic gene expression signatures

The derived 75-probeset Histology signature was compared to published NSCLC histology signatures (Table S9) {[6], [9], [18], [19] and US20040241725A1}. The largest overlap was found with histology signature identified by Garber et al [19], 12 out of 370 genes overlapped with our 75-probeset signature.

The performance of these Histology signatures was tested with our cohort and Duke NSCLC cohort. The correct prediction on the EMC cohort ranged from 56% to 93% (EMC), lower than the 100% correct prediction by our signature. The best performance from published histology signatures on the Duke cohort was 83%, comparable to that produced by EMC Histology signature (84%). ADC-specific signatures performed better when the aimed aggregation was limited to two groups (ADC and non-ADC; Table S9).

**Figure 6:**
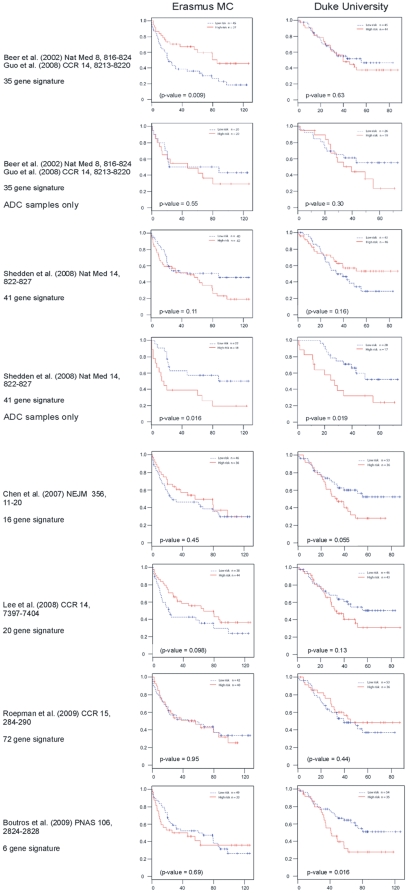
Survival prediction by published prognostic signatures. Kaplan-Meier curves for the best performing signatures (by P-value) are shown for 82 Erasmus MC patients (left) and 89 Duke University NSCLC patients (right), fitted by their risk assignments. Grey bars indicate patients at last follow-up, still alive. P-values are between brackets if overall survival of the low risk group is actually lower than that of the high risk group.

A number of gene expression profiling-derived prognostic predictors have been previously reported for NSCLC [20], [21], [22], [23], [24], [25], [26]. These signatures were derived from a wide variety of platforms and technological approaches (Table S10). We assessed the performance of these previously reported prognostic signatures on the Erasmus MC and Duke University data sets. A total of 14 signatures from 6 different publications were tested (see File S1and Table S10 for details). For each report, the results obtained with the signature yielding the best stratification in low- and high risk groups are displayed in Kaplan-Meier curves (Fig. 6 and Table S10). We find that performance of the 6-gene signature of Boutros et al [26] was reasonable on the Duke University cohort (p-value 0.016) but not on the Erasmus MC cohort (p-value 0.69). The 41-gene signature reported by Shedden et al was developed for ADC samples [21]. Performance of this signature on the complete Erasmus MC and Duke University cohorts was unsatisfactory (p-values 0.113 and 0.158 respectively). However, if the analysis was limited to samples classified as ADC by our histology signature, this was the only prognostic signature that performed well on both cohorts (Erasmus MC p-value 0.016, Duke University p-value 0.019).

The observation that our 75 probe set histology and 17 probe set prognosis signatures perform well on independent cohorts comprising different types of NSCLC suggests that they are robust.

## Discussion

In this study, we defined a set of molecular classifiers for NSCLC. These classifiers were developed with the Erasmus MC cohort of NSCLC patients, and validated using the independent US cohort. The tumor signature gene set can be used to distinguish NSCLC from unaffected lung tissue. The histology signature gene set may aid in the histo-pathological classification of NSCLC in ADC, SCC and LCC. In addition, we identified a survival signature gene set that predicts overall patient survival.

### Potential for improved NSCLC classification

The unique ADC, SCC, and LCC signatures differ in the composition of genes. The ADC signature favors the genes associated with tight junction and cell adhesion molecules. In contrast, SCC signature genes are more correlated with pathways such as cell communication, MAPK, P53, and WNT signaling. Genes included in the SCC signature are NKX2-1, SOX2, FGFR2/3, TP63, PI3K, WNT5A, members of the keratin family, and genes associated with Ras/Rho signaling pathways, such as ERBB2/3. The histological diagnosis of LCC is based on exclusion of the other types of NSCLC. As a result, this subtype of NSCLC is highly heterogeneous in histopathology and clinical presentation. LCC accounts for about 16% of lung cancers. By applying special stains and electron microscopy it has been shown that many cases of LCC are poorly differentiated ADC or SCC (http://www.ncbi.nlm.nih.gov/books/bv.fcgi?rid=cmed.section.20772). The difficulty in distinguishing LCC from other NSCLC by routine histopathology results in considerable variation in the classification of NSCLC cases. In contrast, all molecularly defined NSCLC subtypes share a common gene expression profile which is distinct from the other subtypes. For instance, a number of well-known SCC markers, such as TP63, PERP, Keratins, and SERPINB, were uniformly expressed among a subset of the LCC samples, suggesting that these were actually SCC. In addition, expression profiling revealed that some of the tumors diagnosed as SCC display neuroendocrine characteristics, indicating that these were neuroendocrine tumors and not classical SCC. Thus, the molecular signatures reveal specific features of the tumors. This could be used to improve the classification of NSCLC tumors, especially in histologically heterogeneous tumors where the signatures would identify the most characteristic molecular features of the samples.

This 75-probe set signature conceives molecular characteristics of three histological subtypes, ADC, SCC, and LCC. It is outstanding in information loadage or/and robustness of aggregating NSCLC subtypes than all tested published signatures {[9], [18], [19] and US20040241725A1}.

### A 17- probe set signature set predicts survival

We have identified a small set of survival-associated genes that is able to predict the prognosis independent of histo-pathological tumor type. This novel prognostic profile covers a broad range of NSCLC subypes, and the staging of tumors used for building the prediction model ranged from I to IV (Table 1). Multivariate proportional hazard analysis that included age, smoking years, gender, Forced Expiratory Volume 1, tumor stage, tumor cell content, tumor histology, and tumor size strongly indicates that the gene expression profile-derived prognostic predictor is the strongest predictor of the likelihood of death. Moreover, the performance of these molecular predictors was similar to that in the original dataset in an independent NSCLC patient cohort, indicating its reproducibility and potential clinical relevance. It is possible that the aggressiveness of tumors reflected in this signature is shared by a variety of human cancers. This small set of genes provides potential for application with confidence and practicality required in the clinical setting.

### Divergence of prognostic gene expression signatures

Potti et al [10] developed a metagene model to predict the risk of recurrence for individual patients. The model was predictive for the major types of NSCLC – ADC and SCC, and performed reasonably satisfactory in two independent patient cohorts. Confounding components of the metagene models contain over 100 genes. These attributes complicate the direct comparison of the metagenes to survival signatures derived from other studies. As such, the genes in the metagene model have no predictive power for survival prediction (data not shown).

It has been noted before that there is very little, if any, overlap between the reported prognostic signatures for NSCLC [25], [31]. Remarkably, there is not a single gene shared by the 7 signatures tested here (the 6 best performing previously reported signatures and the 17 probe set signature derived in this paper). This has been attributed to the notion that the space from which such minimized signatures can be derived is large [25], [26] and hence there are many different possible outcomes depending on the particular dataset and bioinformatics approaches taken. In addition, differences between patient populations with respect to ethnic background, tumor histology, smoking status, and other environmental factors may have an impact. For instance, outcome signatures make predictions beyond histological subtype, but it is still possible that genes in the signature are histology-related. When these signatures are applied to other datasets with different tumor composition, they do not necessarily reflect hazard of recurrence or chance of survival. The 41-gene prognostic signature of Shedden et al [21] was developed with ADC samples. We found that stratification of the Erasmus MC and Duke University cohorts by this signature is histology-dependent, since it only performs satisfactorily on the ADC samples in these cohorts. For this analysis, we assigned tumor types in the Erasmus MC and Duke University cohorts with the aid of our histology signature. Thus, a scenario emerges where application of a histology signature is followed by analysis with a tumor type-specific prognostic classifier. Clearly, it is important to test whether prognostic classifiers of NSCLC are operative beyond histological criteria.

**Figure 7:**
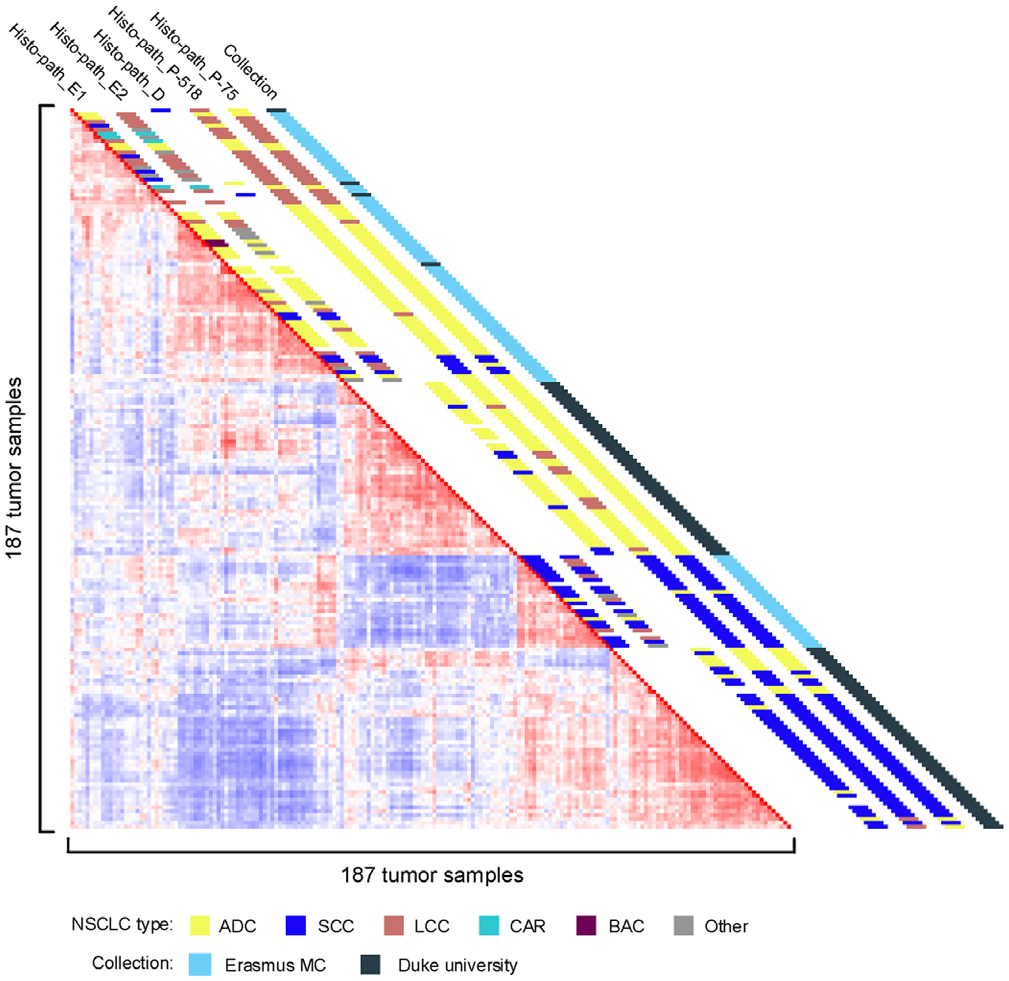
Correlation view of Erasmus MC and Duke University NSCLC samples. In total 187 tumor samples from the Erasmus MC (n = 91) and Duke University (n = 96) cohorts are shown. Pairwise correlations between any two samples are displayed, based on 3495 informative probe sets. Histological classification of the samples, and the collection source, are depicted along the diagonal. The key to the color code is shown at the bottom. Histo-path_E1 & Histo-path_E2: initial and second histo-pathological review of Erasmus MC samples. Histo-path_D: histo-pathological review of Duke University samples; Histo-path_P-518 and Histo-path_P-75: predictions by PAM of histological subtypes using the 518 and 75 probe set signatures, respectively (see Tables S4 and S5).

Alternatively, prognostic classifiers transcending tumor histology would be more straightforward to use. To develop these, different tumor types and subtypes should be included in the experimental set-up. Our dataset covers a relatively broad spectrum of NSCLC, and we have validated the signatures using independent samples profiled using the identical platform [10]. The lack of availability of raw microarray data (.CEL files) precludes validation of our signatures using more independent NSCLC cohorts; the complex issue of cross-platform meta-analysis [22], [32] is beyond the scope of this paper. Nonetheless, our signatures performed well compared to those previously reported [20], [21], [22], [24], [25], [26] when tested using the Erasmus MC and Duke University cohorts. We note that although the Duke University samples are clearly separated from the Erasmus MC samples in unsupervised analysis (Fig. 7) our signatures still perform well on the Duke University data (e.g. Figs. 4B and 5B), indicating that they are robust.

In conclusion, the sets of molecular markers identified in this report reveal histo-pathological attributes of NSCLC. These gene signatures might provide clinically relevant information for NSCLC, transcending traditional histological classification and patient outcome prediction.

## Supporting Information

File S1 Supplementary Materials and Methods.
(0.15 MB DOC)

Table S1 NSCLC Tumor Signature (the longest) T∶N ratio Ratio of average expression in NSCLC samples/normal lung tissue T mean 2log transformation of mean expression value in NSCLC samples (average of all NSCLC and normal lung tissue = 0). N mean 2log transformation of mean expression value in normal lung tissue samples (average of all NSCLC and normal lung tissue = 0). T SD Standard deviation of mean expression value in NSCLC samples N SD Standard deviation of mean expression value in normal lung tissue samples.
(0.21 MB XLS)

Table S2 NSCLC Tumor Signature (long) T∶N ratio Ratio of average expression in NSCLC samples/normal lung tissue T mean 2log transformation of mean expression value in NSCLC samples (average of all NSCLC and normal lung tissue = 0). N mean 2log transformation of mean expression value in normal lung tissue samples (average of all NSCLC and normal lung tissue = 0). T SD Standard deviation of mean expression value in NSCLC samples N SD Standard deviation of mean expression value in normal lung tissue samples.
(0.05 MB XLS)

Table S3 NSCLC Tumor Signature (short) T∶N ratio Ratio of average expression in NSCLC samples/normal lung tissue T mean 2log transformation of mean expression value in NSCLC samples (average of all NSCLC and normal lung tissue = 0). N mean 2log transformation of mean expression value in normal lung tissue samples (average of all NSCLC and normal lung tissue = 0). T SD Standard deviation of mean expression value in NSCLC samples N SD Standard deviation of mean expression value in normal lung tissue samples.
(0.02 MB XLS)

Table S4 NSCLC Histology Signature (long) ADC∶OT ratio Ratio of average expression in ADC samples/the other NSCLC samples (SCC and LCC) SCC∶OT ratio Ratio of average expression in SCC samples/the other NSCLC samples (ADC and LCC) LCC∶OT ratio Ratio of average expression in LCC samples/the other NSCLC samples (ADC and SCC) ADC mean 2log transformation of mean expression value in ADC samples (average of all Erasmus MC samples = 0). SCC mean 2log transformation of mean expression value in SCC samples (average of all Erasmus MC samples = 0). LCC mean 2log transformation of mean expression value in LCC samples (average of all Erasmus MC samples = 0). ADC SD Standard deviation of mean expression value in ADC samples SCC SD Standard deviation of mean expression value in SCC samples LCC SD Standard deviation of mean expression value in LCC samples.
(0.15 MB XLS)

Table S5 NSCLC Histology Signature (short) ADC∶OT ratio Ratio of average expression in ADC samples/the other NSCLC samples (SCC and LCC) SCC∶OT ratio Ratio of average expression in SCC samples/the other NSCLC samples (ADC and LCC) LCC∶OT ratio Ratio of average expression in LCC samples/the other NSCLC samples (ADC and SCC) ADC mean 2log transformation of mean expression value in ADC samples (average of all Erasmus MC samples = 0). SCC mean 2log transformation of mean expression value in SCC samples (average of all Erasmus MC samples = 0). LCC mean 2log transformation of mean expression value in LCC samples (average of all Erasmus MC samples = 0). ADC SD Standard deviation of mean expression value in ADC samples SCC SD Standard deviation of mean expression value in SCC samples LCC SD Standard deviation of mean expression value in LCC samples.
(0.03 MB XLS)

Table S6 NSCLC Patient Survival Signature.
(0.02 MB XLS)

Table S7 Association between the prognostic predictor and clinical parameters.
(0.02 MB XLS)

Table S8 Relation between variables and the relative hazard ratio.
(0.02 MB XLS)

Table S9 Comparison of EMC histology signatures with other NSCLC histology signatures.
(0.02 MB XLS)

Table S10 Comparison of EMC prognostic signatures with other NSCLC prognostic signatures.
(0.02 MB XLS)
