# Supplementary material for: Gene Expression-Based Classification of Non-Small Cell Lung Carcinomas and Survival Prediction
Source: PLoS One. 2010 Apr 22;5(4):e10312. doi: 10.1371/journal.pone.0010312 (PMC2858668; doi:10.1371/journal.pone.0010312)
Supplement: File S1 — Supplementary Materials and Methods. (0.15 MB DOC) [file pone.0010312.s001.doc]

**Supplementary Methods**

*Patient enrolment*

Samples from patients recruited in this study were obtained from two Erasmus MC collections: the Tissue Bank and the Department of Internal Oncology. All lung tumor samples and adjacent non-cancerous specimens were collected from patients who had undergone curative surgical resection between 1992 and 1998 (Internal Oncology), or between 1996 and 2004 (Tissue Bank) at the Erasmus MC. Tissues were collected and studied under an anonymous tissue protocol approved by the medical ethical committee of Erasmus University Medical Center. There were 91 patients with NSCLC included in our analysis. The Tissue Bank and The Department of Internal Oncology obtained written consent from all participants involved in this study.

The study comprised two independent validation sets. The first set included 6 normal lung tissues which were transcriptionally profiled by Affymetrix U133 plus 2.0 array (GSE3526). The second set comprised a cohort of 96 NSCLC patients collected at Duke University, including 50 ADC and 46 SCC samples [1]. Patient characteristics and the original microarray .CEL files were downloaded from <http://data.genome.duke.edu/LungPotti.php>. Eighty-nine out of those samples had relevant follow-up data available and were used for validating the performance of our survival signature.

*Histopathological analysis*

All tumor samples were independently reviewed by two pathologists. The cohort included 32/24 adenocarcinomas (ADC), 27/16 squamous cell carcinomas (SCC), and 13/24 large cell carcinomas. The remaining patients presented with rarer types of lung tumors, such as bronchioloalveolar (BAC), carcinoid (CAR), mixed adeno-squamous, or unknown. In the cohort of patients, over 57 percent had a known smoking history, with an average of 36.7 pack years. Of the 91 NSCLC patients, 51 were at stage I, 21 were at stage II, and 10 were at either stage III or IV. Three patients displayed distal metastases at the time of diagnosis. In addition, eight patients developed multiple primary tumors at different sites originating from the same cell type or different cell types, either synchronously or non-synchronously. Three had undergone neo-adjuvant radiation or chemotherapy before the surgery. Patient and tumor characteristics are listed in Table 1.

*Definining the training and validation sets*

All samples were divided into two subsets, the training set and the validation set, and the former was used to identify NSCLC related molecular signatures.

As a result, thirty-six ‘core’ normal tissues were included in the training set, which showed strong similarities in global gene expression profile with each other and appeared in the core of normal lung cluster in an unsupervised clustering. Tumor samples were divided according to the two independent histopathological reviews, cancer cell contents, and degree of tumor differentiation.

For tumor samples, those from patients with a complete clinical record were assigned into the training set. Samples were excluded from the training set if they fell into anyone of the following cases,

1. From patients who developed multiple primary tumors;
2. Received chemotherapy or radiotherapy prior to the surgery;
3. Tumor cell content < 60%;

To develop histology signatures, additional criteria were employed to create a super training set to sketch a precise histological profile. Tumor samples had to meet below conditions:

1. Consistent classification between two histopathological reviews;
2. No cell type heterogeneity;

As a result, forty-four tumor samples were included in the training set, and twenty-three composed the super training set.

The remaining samples were used as a separate dataset for validating gene signatures identified by the training set. They were either from patients lacking complete clinic information or rejected by the above inclusion criteria, including eight LCC and five of rare types of NSCLC samples with a high level of cell type heterogeneity, and 19 percent (17 out of 91) of tumor samples had a discrepancy in histopathological classification.s

*Total RNA isolation*

The samples used in this study were fresh frozen tissues. Dissected tumors and adjacent normal tissues were snap-frozen in liquid nitrogen precooled isopentane immediately after the surgical resection, and stored at –196°°C or –80 °C until RNA extraction. Specimens were sectioned in Cryostat into slices of 25 µm thick for RNA extraction. For each specimen, two thinner sections (10 µm) were taken at the start and end of collection, and used to determine the percentage of tumor cells. Samples were homogenized with a mortar and pestle in TRI Reagent (Invitrogen, Carlsbad, CA), and then incubated at room temperature for 5 minutes before adding 0.2 µl of chloroform for each 1ml sample. After centrifuging at full speed (12000 rpm) for 20 minutes, the supernatant containing the RNA was precipitated and centrifuged with iso-propanol. The resultant RNA pellets were washed with 75% ethanol and solved in RNase-free water. If applicable, they were stored at –80 °C for further usage.

*Assessment of RNA quality and concentration*

The integrity if the isolated total RNA was verified on the Agilent 2100 BioAnalyzer (Agilent Technologies, Palo Alto, CA). Samples were kept for further processes if the 28s/18s ratio of its RNA was lower than 1.2. The concentrations of the RNAs were measured with a NanoDrop ND-111 UV-VIS spectrophotometer.

*cRNA amplification and labelling*

Double strand (ds) cDNA synthesis was performed according to the standardized protocol for One-Cycle cDNA synthesis from Affymetrix (Santa Clara, CA). Approximately 5 µg of total RNA was first converted to single strand cDNA in a 20 µl First-Strand Reaction Mix, containing poly-A control RNA, 100 µmol T7-Oligo Primer, 1x first strand buffer, 0.2 mol DTT 10 mmol dNTP mix and SuperScript II. In detail, the sample RNA, the poly-A control RNA and the T7-Oligo Primer were mixed and incubated for 10 min at 70 °C. Secondly, the first strand buffer, the DTT and the dNTP mix were added and incubated for 2 min at 42 °C, followed by adding SuperScript II and incubation of 1 hour at 42 °C. The ds cDNA was prepared from the resultant First-Strand Reaction Mix, mixed with 1x second strand reaction buffer, 30 mmol dNTP mix, E.coli DNA ligase, E.coli DNA Polymerase I and RNaseH. The mix was incubated for 2 hours at 16 °C, then supplemented with T4 DNA Polymerase, and then incubated for another 5 minnutes at 16 °C. The reaction was stopped by the addition of EDTA to a final concentration of 5 µM. The Sample Cleanup Module and GeneChip IVT Labeling Kit from Affymetrix were used to purify the synthesized ds cDNA, which was used to generate biotin-labeled cRNA, in the presence of 1x IVT Labeling buffer, IVT Labeling NTP Mix, IVT Labeling Enzyme Mix and RNase-free water in a total volume of 40 µl. After an incubation of 16 hours at 37 °C, the concentration and quality of the labelled cRNA were checked with NanoDrop ND-1000 UV-VIS spectrophotometer. An A_260_/A_280_ ratio between 1.9 and 2.1 was considered acceptable. Approximately 20 µg cRNA per array was fragmented to an average size of 35-200 nucleotides by heating at 94 °C for 35 min, in the presence of a 1x Fragmentation Buffer in a total volume of 40 µl. The undiluted, fragmented samples were stored at –20 °C before being subjected to hybridization.

*Hybridization*

Hybridization was conducted following Affymetrix instruction for GeneChip® Human Genome U133 plus 2.0 array. The GeneArray scanner 3000 (Affymetrix) was then employed to detect the hybridization signals.

***Preprocessing microarray data***

*Array Quality Control*

Microarrays that did not pass the quality assessment were removed from further analyses. The quality metrics used to exclude microarrays was the statistics summary calculated by the GCOS algorithm during the processing of probe-level data. The primary inclusion criteria include: all arrays had to have comparable noise values (Raw Q, measurement for the pixel-to-pixel variation of probe cells on the chip); background values were within the range of 20 to 100; percent of present probe sets on the array should not be below 45%. The other criteria were: arrays with extremely high or low values for any of these parameters, e.g. values beyond the range of standard deviation ± median, were excluded; signal ratio of ≤3 of the 3’ / 5’ probe sets for GAPDH and Actin were used as a cut-off; labelling and hybridization were controlled by using standard spike-in controls according to the Affymetrix protocol; if global scaling was applied, the scaling factors for each array were within a three-fold range.

***Array Data analysis***

Microarray data was processed at two levels: probe level and probe set level.

*At probe level by quantile normalization*

RMA (Robust Multi-Array average) is an integrated algorithm comprising background adjustment, quantile normalization, and expression summarization by median polish [2]. The intensities of mismatch probes were entirely ignored due to their spurious estimation of non-specific binding. The intensities were background-corrected in such a way that all corrected values must be positive. The RMA algorithm utilized quantile normalization in which the signal value of individual probes was substituted by the average of all probes with the same rank of intensity on each chip/array. Finally Tukey’s median polish algorithm was used to obtain the estimates of expression for normalized probe intensities. All raw data and RMA normalized data are MIAME compliant and available in a MIAME compliant database, Gene Expression Omnibus database at www.ncbi.nlm.nih.gov/geo/info/linking.html (GSE19188).

*At probe set level by Global Scaling (GCOS v1.4)*

This algorithm was a summary method embedded in GeneChip Operating Software (GCOS) from Affymetrix, and fully described in the data_analysis_fundamentals_manual. The signal intensity of each probe was firstly corrected by the overall background. The differences between perfect match (PM) and mismatch (MM) probes were examined by using background-adjusted intensities for each probe pair. The significance of the differences between PM and MM probe sets was reflected by a p-value calculated by one-sided Wilcoxon-signed rank test. The final signal for a probe set was assigned as the one-step biweight estimate of the combined differences of all probe pairs belonging to one probe set. The trimmed mean signal of each array was then scaled to the same Target Intensity (e.g. 250) by a global method to minimize technique-derived discrepancies.

*Other transformations*

Intensities of probe sets lower than 30 were reset to 30. The geometric mean for each probe set was calculated across all samples or for each subgroup of samples firstly and then across all samples (OmniViz). The intensity values of individual probe sets in each sample were then displayed as the log 2 of the deviations to the calculated geometric means.

*Probe sets filtering*

Probe sets were involved in further analysis only if their expression levels deviated from the overall mean in at least one array by a minimum factor of 2.5, because the remaining data were unlikely to be informative. The result was that 43,160 probe sets were eliminated, and 11,515 probe sets remained for further analysis.

1. Unsupervised clustering and visualization of gene/sample similarity

Clustering was performed without taking into account any external information such as histology subtypes and tumor stages, with each of the selected 11,515 probe sets using the K-means algorithm (OmniViz). Similarities were measured by magnitude and shape (Euclidean distance). Pair-wised similarities between samples were sorted and visualized by the Pearson Correlation Matrix (OmniViz). The order of clusters and individual samples within each cluster was sorted according to the Pearson Correlation Coefficient.

***Statistical analysis***

The resulting 11,515 probe sets from the filtering step was the starting point for all supervised analyses which, for instance, correlated gene expression with the clinical variables such as the histological subtype. Two-Class comparison analysis was performed by using Significance Analysis of Microarray (SAM), integrated in OminiViz version 5.1. Class prediction analysis was performed with the use of Prediction Analysis of Microarrays (PAM) software, integrated in BRBArray version 3.8. Clustering was performed using the Spotfire DecisionSite software (TIBCO, Palo Alto, CA). The samples were clustered with various signatures using the Weighted Pair-Group Method algorithm and similarity measured by Euclidean distance or correlation. Samples were assigned as unpaired for SAM and PAM analyses.

*Class comparison*

SAM discovered differentially expressed genes among different sample classes, e.g. between non-cancerous tissues and tumors or between a particalur histology subtype and the remaining samples [3]. First, we used the training set to compare each histological type of tumors separately with corresponding healthy tissues. Next, all training set tumors were combined as one group and compared to the group of all training set healthy lung tissues.

The SAM algorithm calculated the different expression for each gene between classes relative to the variation expected in the mean difference. To correct multiple testing, false discovery rate (FDR) was controlled by randomly permutating the classes of samples 100 times. Signature probe sets for assigned classes were selected by a change factor of 2. Different selective cut-offs of FDR were used which generated lists of differentially expressed probe-sets ranging from thousands to hundreds. The cut-off which produced the shortest list and the lowest FDR was used. All class comparisons were performed with both RMA- and GCOS-processed data. The common probe sets identified by both sets of data were selected as the final signatures.

*Class prediction*

The resultant signatures from Class Comparison were tested by the nearest shrunken centroids algorithm (PAM) to identify subgroups of genes that best characterized the predefined classes [4]. The prediction accuracy of optimized signatures was determined by performing “leave-one-out” cross validation within the training set, with one sample omitted each time and class label being predicted with other samples for the omitted sample [5]. The predictive models generated by the optimal subsets were subsequently applied to make predictions of classes for samples in the validation set, which were not involved in the corresponding class comparisons. The prediction accuracy on validation samples was calculated by comparing predicted class labels with the clinical histopathological diagnoses for those samples; samples without histopathological records were excluded from the calculations.

***Survival analysis***

Of 91 NSCLC samples, 82 have relevant follow-up data available. Therefore, those samples were included in survival analysis.

Two different approaches were used to determine whether the gene expression profile could predict the prognosis for NSCLC patients. In one approach, samples from patients who died of lung cancer within two years of surgical removal of tumors were assigned to the group of NSCLC with short-time survival. The long-time survival group consisted of samples from patients who survived for longer than 5 years. To avoid unexpected variances introduced by the failure of surgery or postoperative sequelae, those patients who died within six months of surgery were kept out of the analysis. Subsequently, the same analysis was performed conditionally for histological subtypes.

As an alternative way, we developed a step-wise approach based on gene expression profiles to classify NSCLC with respect to prognostic outcome. Firstly, probe sets which were the most likely associated with patient prognosis were selected among over 11,000 probe sets by their correlation with the defined survival time; A list of candidate probe-sets was created with probe-sets whose univariate p-values, testing the hypothesis that survival time is independent of the expression level for that gene, was smaller than 0.001 by the Wald test in the Cox proportional hazards model [6]. A global test was performed with 1000 permutations to adjust p-values.

In the analysis of the probability that patients would remain free of death, survival time (OS) was defined as the date of surgery to the time of event happened – death, or the date on which data were censored - the last follow-up visit.

The resulting candidate survival probe-sets were subjected to a supervised principal component calculation described in details by Bair et al [7]. The computation of principal components was followed by Cox proportional hazards regression analysis using the computed principal components. As a result, a predictive prognosis model for NSCLC was determined, with regression coefficients derived from the Cox regression described above. With the developed model, a prognostic predictor was calculated for a NSCLC case whose expression profile was provided as the expression levels of selected probe-sets.

The predictive value of the prognosis model was evaluated by performing “leave-one-out” cross-validation”, in which a single case was omitted each time and the entire procedure described above was performed to estimate prognosis predictor for the omitted case [7,8]. This prognosis predictor value was compared and ranked relative to the prognosis predictors of cases included in the cross-validation training cases. Based on the predetermined cut-off percentile rank for defining the risk groups, the omitted case was placed into a risk group. This analysis was repeated until each sample was left out once, resulting in a set of unbiased prognosis prediction for all cross-validated samples.

Having obtained unbiased prognosis predictors and consequent categorizing patients, the difference in the survival outcome between risk groups was estimated by log-rank Mantel-Cox test and plotted by Kaplan-Meier curve [9]. The analyses were performed with BRB-Array Tools (version 3.8; R.Simon and A.P.Lam, National Cancer Institute, Bethesda, MD).

To evaluate the prognostic value of the prognosis predictor relative to other clinical parameters, we used proportional hazard regression analysis with the defined survival time as dependent variable, death as the occurred event, and the last follow-up visit as the censored. The risk of death studied included age, tumor cell content (%), tumor size (diameter of tumor), smoking year, Forced Expiratory Volume 1, and gender, tumor histology, tumor grade, as well as computed prognosis predictor. The relation between them and the relative hazard ratio was tested with use of the Wald test (Table S8). To compare the performance in predicting the overall OS, the proportional hazard regression model was built with either involving a specific parameter or not. The contribution of each parameter to the model was evaluated by chi-square test and P-value was derived from the likelihood ratio test (Table 2) [6].

The correlation between the survival signature and clinical parameters was evaluated using predicted risk as grouping variable and with independent samples t-test for continuous variables, or non-parametric test, Mann-Whitney and maximum possibility Wald-Wolfowitz test, for categorical variables and scalar variables (Table S7). Statistical analyses were performed with SPSS 15.0 (SPSS, Chicago, IL). For each tumor from NSCLC validation cohort, we calculated a prognosis predictor by fitting the predetermined predictive model with expression of the 17 probe sets. Patients were predicted with high-risk of death if their prognosis predictor percentile ranking was above the 60^th^, as determined in the procedure of identifying prognosis signature using training samples.

***Comparison with published prognostic signatures***

1. ***Prognostic signatures***

If the original prognostic predictors were provided as gene symbols [10,11,12,13,14], we retrieved gene expression for the Erasmus MC and Duke University cohorts as follows. First, genes were mapped to the Affymetrix U133 plus 2.0 chip, and the corresponding expression data from all relevant probe sets was extracted (Table S10). Next, probe set level data was converted to gene level data by averaging probe sets targeting the same genes. Due to the variation between platforms, 4 genes from the Roepman et al [13] signature were missing from the Affymetrix U133 plus 2.0 chip, we used the remaining 68 genes.

When the original prognostic predictors were supplied as probe sets [15,16], either from Affymetrix U133A or U133 plus 2.0 arrays, the data was kept at probe set level. The Affymetrix HuGeneFL chip used by Beer et al / Guo et al [15,16] deviates too much from the U133 plus 2.0 chip and we therefore used gene symbols to re-map the data to the U133 plus 2.0 chip.

Some studies provide multiple signature sets [11,15,16], in which case each signature set was tested. For all re-evaluations, a cut-off at the 50th and 60th percentile was used for dividing the two risk groups. We only show the results for the best stratification obtained (Fig. 6 and Table S10).

1. ***Histology signatures***

The retrieval of expression data of signature genes was same as described in the previous section. The reproducibility of previously published histology signatures was assessed using both Erasmus MC and Duke NSCLC cohorts {[17,18,19] and US20040241725A1}. Different predictive algorithms were used. For EMC NSCLC samples, the signatures were applied to aggregate three major subtypes, ADC, SCC, and LCC. In case the signature is devoted to a specific subtype, such as ADC [17,19], it was subsequently applied to cluster NSCLC into two classes, ADC and non-ADC. All signatures were applied to cluster Duke NSCLC samples into two classes, ADC and SCC. The correct prediction rate was calculated by comparing the predicted Histology to the gene-assigned Histology (EMC) or the pathological review (Duke). The results from 1-Nearest Neighbour algorithm which successfully classified all tumor samples in EMC and Duke cohorts are shown in Table S9.

**References**

1. Potti A, Mukherjee S, Petersen R, Dressman HK, Bild A, et al. (2006) A genomic strategy to refine prognosis in early-stage non-small-cell lung cancer. N Engl J Med 355: 570-580.

2. Irizarry RA, Hobbs B, Collin F, Beazer-Barclay YD, Antonellis KJ, et al. (2003) Exploration, normalization, and summaries of high density oligonucleotide array probe level data. Biostatistics 4: 249-264.

3. Tusher VG, Tibshirani R, Chu G (2001) Significance analysis of microarrays applied to the ionizing radiation response. Proc Natl Acad Sci U S A 98: 5116-5121.

4. Tibshirani R, Hastie T, Narasimhan B, Chu G (2002) Diagnosis of multiple cancer types by shrunken centroids of gene expression. Proc Natl Acad Sci U S A 99: 6567-6572.

5. Golub T, Slonim D, Tamayo P, Huard C, Gaasenbeek M, et al. (1999) Molecular classification of cancer: class discovery and class prediction by gene expression monitoring. Science 286: 531-536.

6. Cox DR (1972) Regression models and life-tables. J R Stat Soc 34: 187-220.

7. Bair E, Tibshirani R (2004) Semi-supervised methods to predict patient survival from gene expression data. PLoS Biol 2: E108.

8. Simon R, Radmacher MD, Dobbin K, McShane LM (2003) Pitfalls in the use of DNA microarray data for diagnostic and prognostic classification. J Natl Cancer Inst 95: 14-18.

9. Meier P, Kaplan E (1958) Nonparametric estimation from incomplete observations. J Am Stat Assoc 158: 457-481.

10. Boutros PC, Lau SK, Pintilie M, Liu N, Shepherd FA, et al. (2009) Prognostic gene signatures for non-small-cell lung cancer. Proceedings of the National Academy of Sciences 106: 2824-2828.

11. Chen HY, Yu SL, Chen CH, Chang GC, Chen CY, et al. (2007) A five-gene signature and clinical outcome in non-small-cell lung cancer. N Engl J Med 356: 11-20.

12. Guo NL, Wan Y-W, Tosun K, Lin H, Msiska Z, et al. (2008) Confirmation of Gene Expression-Based Prediction of Survival in Non-Small Cell Lung Cancer. Clin Cancer Res 14: 8213-8220.

13. Roepman P, Jassem J, Smit EF, Muley T, Niklinski J, et al. (2009) An immune response enriched 72-gene prognostic profile for early-stage non-small-cell lung cancer. Clin Cancer Res 15: 284-290.

14. Beer DG, Kardia SL, Huang CC, Giordano TJ, Levin AM, et al. (2002) Gene-expression profiles predict survival of patients with lung adenocarcinoma. Nat Med 8: 816-824.

15. Lee ES, Son DS, Kim SH, Lee J, Jo J, et al. (2008) Prediction of recurrence-free survival in postoperative non-small cell lung cancer patients by using an integrated model of clinical information and gene expression. Clin Cancer Res 14: 7397-7404.

16. Shedden K, Taylor JM, Enkemann SA, Tsao MS, Yeatman TJ, et al. (2008) Gene expression-based survival prediction in lung adenocarcinoma: a multi-site, blinded validation study. Nat Med 14: 822-827.

17. Bhattacharjee A, Richards WG, Staunton J, Li C, Monti S, et al. (2001) Classification of human lung carcinomas by mRNA expression profiling reveals distinct adenocarcinoma subclasses. Proc Natl Acad Sci U S A 98: 13790-13795.

18. Garber ME, Troyanskaya OG, Schluens K, Petersen S, Thaesler Z, et al. (2001) Diversity of gene expression in adenocarcinoma of the lung. Proc Natl Acad Sci U S A 98: 13784-13789.

19. Kobayashi K, Nishioka M, Kohno T, Nakamoto M, Maeshima A, et al. (2004) Identification of genes whose expression is upregulated in lung adenocarcinoma cells in comparison with type II alveolar cells and bronchiolar epithelial cells in vivo. Oncogene 23: 3089-3096.
